# Does Part-Time Mothering Help Get a Job? The Role of Shared Custody in Women’s Employment

**DOI:** 10.1007/s10680-022-09625-4

**Published:** 2022-06-27

**Authors:** Carole Bonnet, Bertrand Garbinti, Anne Solaz

**Affiliations:** 1 The French Institute for Demographic Studies (INED), Aubervilliers, France; 2 CREST–ENSAE-Institut Polytechnique Paris, Paris, France

**Keywords:** Shared custody, Employment, Divorce, Lone mother, Separation, Child arrangement

## Abstract

Though child shared physical custody arrangements after divorce are much more frequent and parents who use it more diverse in many European countries, little is known about their economic consequences for parents. By relaxing family time constraints, does shared custody help divorced mothers return to or stay on work more easily? Since lone mothers are one of the least-employed groups, and they face high unemployment rates, the type of child custody arrangement adopted after divorce is of particular interest for their employability. This article analyses to what extent the type of child custody arrangement affects mothers’ labour market patterns after divorce.

Using a large sample of divorcees from an exhaustive French administrative income tax database, and taking advantage of the huge territorial discrepancies observed in the proportion of shared custody, we correct for the possible endogeneity of shared custody. Results show that not repartnered mothers with shared custody arrangements are 24 percentage points more likely to work one year after divorce compared to those having sole custody, while no significant effect is found for repartnered mothers. Among lone mothers, we also highlight huge heterogeneous effects: larger positive effects are observed for previously inactive women, for those belonging to the lowest income quintiles before divorce, for those with a young child, and for those who have three or more children. Thus, shared physical custody arrangements may reduce work–family conflict by diminishing childcare expenses and enlarge the possibilities to find a suitable job because of more relaxed time constraints for lone mothers.

## Introduction

While sole custody with the mother remains the most frequent arrangement after divorce, the proportion of parents who adopt a shared physical custody arrangement has substantially increased in many countries over recent years (Cancian et al., 2014). By shared custody, we refer here to child physical custody, that is to say, the child living arrangements and not shared (or joint) legal custody, which concerns arrangements for decision-making about parenting such as child’s health, education, and property. This means an equal or roughly equal division of time the child spends with each parent. Although cross-national comparisons should be considered with caution because of non-consistent definitions across countries and data sources (Smyth, 2017), the proportion of recent divorces with shared custody arrangements has reached one out of five separations in many European countries such as France (21%, Carrasco & Dufour, 2015), the Netherlands (22%, Poortman & van Gaalen, 2017), and Spain (28%, Solsona & Ajenjo, 2017), while it represents one third or more of divorces in Belgium (33%, Sodermans et al., 2013a; Sodermans et al., 2013b, 37%, Vanassche et al., 2017), Sweden (Statistics Sweden, 2014), and Norway (Kitterød & Wiik, 2017).

Shared custody was commonly practiced by mostly a small, selected group of socio-economically advantaged parents who had separated, but the families now concerned are more diverse (Kitterød & Wiik, 2017; Meyer et al., 2017). The increasing frequency of joint custody arrangements might be linked to changing parenting norms, typically with fathers spending more time with their children in recent decades (Gimenez-Nadal & Sevilla, 2012; Hook 2016). New laws promoting co-parenting have also encouraged parents to share equally their parental responsibilities and time spent with children after their separation. In several European countries (Spijker & Solsona, 2016) and in the USA, initiatives have been implemented to make joint physical custody the default or legally presumed post-divorce arrangement, and this has sometimes sparked vigorous debates such as recently in France.

Though shared custody arrangements after divorce have become more and more frequent, little is known about their economic consequences for parents, particularly in terms of employment. Finding a job or remaining at a current one are ways to avoid or reduce losses in disposable income after divorce. As such, this, in turn, helps separated mothers and their children escape poverty, which is a huge risk in many countries (Bradshaw and et al., 1996, Brady & Burroway, 2012).

However, re-entering or remaining in the labour market after divorce could be hindered by the presence of children. Since children’s post-divorce living arrangements are a potential source of heterogeneity in a mother’s employment behaviour, the post-divorce effects of these arrangements need to be addressed. Here, we focus on mothers’ post-divorce labour market participation, because mothers more frequently bear the negative consequences of break-ups (Bianchi et al., 1999; Finnie, 1993; Smock, 1994) and are more at risk of poverty (Ananat & Michaels, 2008). By relaxing childcare constraints and improving the work–family balance of lone mothers after separation, labour market participation may be supported by shared custody.

Using rich French administrative fiscal data with precise information on child custody arrangements at the individual level as well as on many other covariates, this paper aims to analyse whether shared custody leads recently divorced mothers to remain in the labour market or to re-enter if they had previously withdrawn during their marriage. Our identification strategy uses both individual-level data on child custody arrangements and territorial discrepancies in the proportion of shared custody arrangements to correct for the possible endogeneity of shared custody arrangements after a divorce. We find that a mother’s employment rate increases by 22 percentage points on average (24 pp for not repartnered) when the parents opt for a shared custody arrangement compared to those having sole physical custody. Larger positive effects are observed for more disadvantaged mothers in the labour market. More specifically, we show particularly large effects for previously inactive women, women belonging to the lowest income quintiles before divorce, women with a young child, and mothers who have three or more children.

Our contribution is fourfold. First, it creates a bridge between the literature on the economic consequences of divorce, custody reforms, and mothers’ labour supply. Second, we are able to use individual-level measures of post-divorce life-course arrangements and directly study our population of interest: divorced mothers with a shared custody arrangement. It contrasts with previous economic studies on shared custody (Böheim et al., 2012; Halla, 2013; Vuri, 2018) that use aggregate measures or changes in the law, and consider people at risk of divorce (the whole population or married women) or divorcees regardless of the kind of childcare arrangements. Our third contribution is a focus on de facto situations (joint physical custodies) rather than on legal arrangements (joint legal custodies). The de facto arrangements are more informative about the time each parent spends with her/his child. Because they contribute to daily life schedules, the de facto arrangements have far more consequences on parents’ labour market outcomes than do legal arrangements.^1^ Fourth, we tackle selectivity or reverse causality issues in shared custody arrangements using local disparities in shared custody prevalence. Fifth, our study provides recent original empirical evidence on the economic consequences of shared custody in a changing European context of post-divorce arrangements, whereas the previous economic literature has mainly focused on the USA.

## Background

### Related Work

Few empirical studies are able to isolate the effect of joint custody from other arrangements. Most research on the consequences of post-divorce custody arrangements is primarily sociological or demographical, with a focus on children outcomes (Nielsen, 2014). They consider diverse dimensions such as child educational attainment, behaviour, health, stress (Turunen, 2017) or well-being (Bauserman, 2012; Vanassche et al., 2013). Regarding the consequences for adults, the literature focuses on non-economic consequences in terms of conflict (see Steinbach’s, 2019 review), repartnering opportunities (Berger et al., 2018; Ivanova et al., 2013; Schnor et al., 2017), well-being (Sodermans et al., 2015), and health (Melli & Brown, 2008, Struffolino et al., 2016).

Some recent economic works have estimated the causal effects of shared custody on diverse economic outcomes such as working participation and hours worked. Most of them use variations in the timing of different reforms of legal shared custody arrangements across the USA. In this line, Vuri (2018) studies changes in the labour market outcomes of single mothers in the USA following legal child custody reforms, showing that shared custody introduction at the state level has no effect on the probability of being in the labour force. Several other articles have previously used the same identification strategy, but with the exception of Vuri (2018), they estimate either an effect on currently married parents only in a household bargaining power approach (Altindag et al., 2017, Nunley and Seals, 2011) or an overall effect on a whole population that mixes both currently married and divorced people (Halla, 2013). It is thus difficult to infer an effect on divorced mothers (since married women numerically dominate divorced one). Indeed, as hypothesized by Halla (2013), the effects of shared custody regime on employment might differ for married and divorced mothers: while the shared custody regime has unclear effects for married women, possible positive effects are assumed to exist for divorced women, since they may spend less time on parenting. Halla (2013) is, however, unable to test this assumption with the data he used.

Employment after divorce has been indirectly studied by two branches of the literature. First, since lone parents and particularly lone mothers are at a higher risk of poverty and unemployment, they were particularly targeted by activation policies (also called “Active Labour Market Policies” (Whitworth, 2013)) that aim to increase their employability and earnings capacities, notably by strengthening the work incentives. The welfare policy literature in several countries (Francesconi & van der Klaauw, 2007; Gregg et al., 2009, for UK; Meyer, 2002, for USA; Dang & Trancart, 2011, for France) found a positive effect from such activation policies on lone parents’ employment rates at the extensive or intensive margins. However, because the control groups they use are either parents in a relationship or single and childless women, they are not able to measure possible differentiated effects of post-divorce child arrangements on a divorced mother’s employability. Thus, they implicitly assume that mothers have sole custody, which was a reasonable hypothesis as long as the mother with sole custody was by far the dominant model. This can be called into question as shared custody arrangements become more widespread.

Second, another group of studies highlights the crucial role played by childcare costs in a mother’s employment probabilities. For instance, Goux and Maurin (2010) find a positive effect of early school availability on a lone mother’s employment. Francesconi and van der Klaauw (2007) show that the working family tax credit program aiming at encouraging work among low-income families has a higher positive impact on mothers with one pre-school-aged child. However, the difficulties faced by single mothers in obtaining or keeping a job after divorce may also differ depending on parental arrangements for the children (whatever their age), a dimension that has been largely neglected in the literature. The research and policies have stressed the importance of employment in helping single mothers out of poverty, placing special emphasis on the childcare issue as a crucial determinant of their employability. However, most articles have not been able to take into account post-divorce arrangements at the individual level. More specifically, among lone parents, they do not distinguish between those with full-time (or nearly full-time) children from those in shared custody arrangements who share their childcare time more equally.

### How can Custody Arrangements Affect a Mother’s Activity?

Several mechanisms may explain how the type of custody arrangement after a divorce can affect mothers’ activity.

First, as previously mentioned, time availability is a crucial point. In terms of childcare, shared custody arrangements are less time-consuming for parents than are sole custody arrangements. As mothers may spend less time on parenting activities, they may increase time devoted to other activities such as work and possibly leisure (Van der Heijden et al., 2016). Balancing work and family (Van der Heijden et al., 2016) and pursuing careers (Kitterød & Wiik, 2017) may be easier if mothers are able to work more intensively one out of two weeks. Thus, shared custody might thus help mothers continue working or enter a new job.

Second, an income effect may exercise its role in two opposite directions. On the one hand, children’s needs are “equally shared” more naturally and child costs are balanced between parents in case of shared custody. Thus, mothers who are granted shared custody might need less money for their children than those with sole custody.^2^ This could negatively affect her likelihood to work. On the other hand, divorces with shared custody arrangements (at least in France) are associated with either no child support payments or with considerably lower amounts than those received through sole custody (Sayn et al., 2012). Parents are generally considered to share equally child costs because they share equally parental time. This absence (or lower amount) of child support payment may be an incentive for mothers to work more, since public transfers for lone mothers only partially alleviate budget constraints. However, part of the mothers in sole custody arrangements do not necessarily receive child support payments either because the father does not pay them or because no decision has been made (Lardeux, 2021). We observe in our data a socio-economic status -gradient in child custody payments (see appendix A1), both in terms of prevalence and amount received. Mothers in low-income households receive less often and lower amounts than mothers in more affluent households, even though a state-funded transfer (named “Allocation de Soutien Familial”—family support allowance) may compensate the non- or partial payment in certain cases. It thus means that the loss in father payments in case of shared custody is not systematic and does not have the same magnitude along the income distribution. It may involve heterogeneous effects by sub-groups.

Third, job market opportunities might be reduced in case of shared custody because of the job search area being reduced. Due to the child frequently commuting between parental homes, parents are constrained to living near each other as well as near their child’s school (Stjernström & Strömgren, 2012; Thomas et al., 2018; Ferrari et al., 2019). For this reason, parents granted shared custody are less likely to accept a job far from their home than would parents with sole custody.

Lastly, we should note that parents who are granted shared custody arrangements are selected, though less so than some decades ago. They are generally more economically advantaged. They may thus have different (probably lower) needs to work because of their savings, but they may also have different preferences towards work (e.g. being more work-oriented, for instance). They could also have more egalitarian values about sharing parental care. Divorces with shared custody arrangements are generally less conflictual (Kitterød & Wiik, 2017),which might facilitate parents experiencing improved self-esteem and attitudes towards work. This may also help mothers become less stressed and thus recover more easily after divorce, which in turn will facilitate their maintaining or re-entering the labour market. This potential selection issue is also a crucial point to take into account.

To summarize, the overall effect of shared custody on women’s labour market outcomes is unclear and depends on the relative strength of various effects. Moreover, the time constraints and the economic pressure may differ according to mothers’ characteristics. For instance, for mothers having young or several children, shared custody may particularly relax the time constraints compared to sole custody. The economic pressure to work may be stronger for mothers with low financial resources. Although we are not able to disentangle time from income mechanisms, we consider potential heterogeneous effects of shared custody depending on several dimensions: the number of children and the age of the youngest child, the pre-divorce household income, and the working status prior to divorce.

## French Context

### Employment Rate of Lone Mothers

Lone mothers with young children are one of the least-employed groups, and they face high unemployment rates. Separated women with young children and/or several children may face difficulties in returning to the labour market because of family–work schedule conflicts. As Table 1 shows, French lone mothers are more willing to be in the labour force than are mothers in a relationship, whatever the number of children. However, lone mothers are actually less often employed than mothers in a relationship. This lone motherhood penalty on job access may partly come from their greater difficulties in balancing family and work. For instance, they may be more likely to decline jobs with demanding schedules or those that require long-distance commuting. Note also, however, that when they are employed, they are more often working on a full-time basis, probably because of heavy financial constraints.

**Table 1:** Mothers’ labour force participation in France, 2004–2007 (%)

	In a relationship	Lone mothers
Labour market participation rate		
All mothers	82.7	88.5
1 child	89.1	92.3
2 children	84.8	88.9
3 children or more	66.2	72.6
Employment rate		
All mothers	73.5	70.2
1 child	79.7	75.5
2 children	76.5	70.6
3 children or more	55.5	48.9
Part time among workers		
All mothers	35.0	26.8
1 child	26.5	22.9
2 children	38.4	29.7
3 children or more	47.0	40.2

INSEE, annual census surveys from 2004 to 2007* (*Chardon & Daguet, 2008*)*

### Child Living Arrangements Decisions after Divorce

In cases of divorce with children, parents in France are required to define their type of custody arrangement. Child custody arrangements in France are generally decided by the parents on the advice of lawyers and submitted for the approval of a family court judge. To assess a parental request for shared custody, family court judges are asked to take into account the child’s best interests.^3^ They evaluate this through several dimensions: the child’s age and maturity, the relationship between parents, the distance between the parental homes, and other general characteristics of the situation (parents’ availability, comfort of the dwellings, etc.). There are no specific rules about how to consider and weight each of these elements, and they are therefore open to interpretation. In practice, judges rarely go against the parents’ request. In most cases (90% of cases, according to the Ministry of Justice (Guillonneau & Moreau, 2013), the parents relied on the help of their lawyers to agree on custody before judgment, which guarantees a quicker process.

**Fig. 1:**
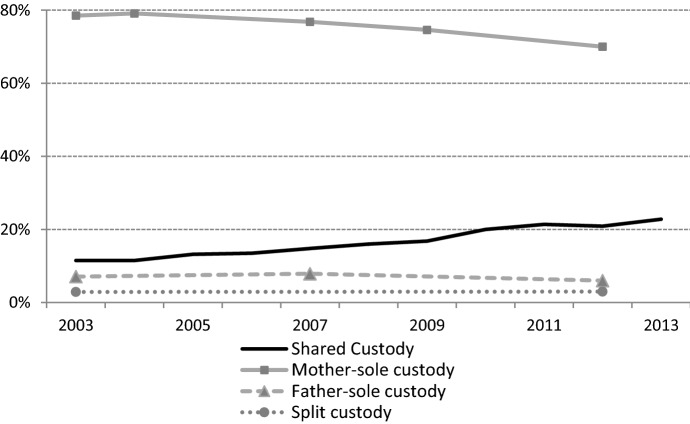
Trends in post-divorce arrangements in France, 2003–2013. Source: Data from the Ministry of Justice. See Chaussebourg and Baux (2007), Chaussebourg et al. (2009), Belmokhtar (2014). Data are not available after 2013. *Note* Split custody concerns families with several children who adopt different post-divorce arrangements, depending on the child

We observe a sharp increase in shared custody arrangements from 2003 on (Fig. 1), even though the most common arrangement is still sole custody with the mother. While the proportion of mothers with sole custody has been decreasing, the proportion of parental divorces followed by shared custody arrangements has doubled in less than 10 years. It concerns more than one out of five divorces involving children in 2013 (Bonnet et al., 2015). The family judge also decides on the opportunity and amount of the child custody payment. In cases of shared custody, child custody payments are less often decided (23%) than in cases of mothers with sole custody (83%) (Carrasco & Dufour, 2015).

### Welfare Benefits System

Welfare programs may mitigate the negative economic consequences of divorce for mothers. First, lone parents can benefit from social benefits (RSA,”revenu de solidarité active”) that depend on the household characteristics (number of children, single versus couple) when they are not working. They benefit from substantial tax reductions associated with children and, as other parents, from family allowances. Lone parents are also given strong incentives to enter and return to the labour market thanks to an additional in-work component, attributed to working individuals (see Bozio et al, 2020 for more details). As a result, it is not clear whether the perception of welfare benefits could constitute a strong disincentive to work, and whether public transfers may play differently for mothers with sole or shared custody.

Indeed, as pointed by Meyer & Carlson, (2014), public benefits are not always or are only partially adapted to shared custody arrangements. For instance, in France, while family allowances and tax discounts associated with children (“quotient familial”) may be shared in case of shared custody since 2007, for housing allowances the law was only voted in 2017 but has been only partially implemented so far. There is no possibility to share childcare subsidies.

## Data, Sample, and Outcome

As recently pointed by Bernardi and Mortelmans (2021), the lack of good and representative data on shared custody arrangements is an issue. Though crucial, the individual information on child custody arrangements is often missing or concerns too few cases in usual surveys to study its consequences precisely. That is why we use an administrative and exhaustive income-tax returns database that has been recently made available in France. We selected a sample of working-age mothers who divorced in 2009 (and also mothers who broke their civil partnership called PACS), whom we can observe the year before and the year following the break-up. We thus have information about the family structure, individual incomes of each spouse, and residence before and after separation. In contrast with usual survey data, we are able to follow the vast majority of the individuals (even when they move). Nevertheless, around 12% of divorcees are not recovered after divorce. They are either lost (migration or death), unable to be matched with pre-divorce information, or one of their tax returns (income or housing tax) is not recovered. Thanks to the exhaustive information on pre-divorce characteristics,^4^ we have been able to compute weights to ensure representative results at the national level. After excluding missing data and residents of overseas territories (for whom some contextual data were missing), we obtain a representative sample of 60,700 mothers aged 20 to 55 who were married or “PACSed” (in 2008) and separated in 2009.

The huge size of our dataset allows observing a substantial number of parents with shared custody arrangements (9,646), which contrasts with most previous studies using survey samples. The custody arrangement should be reported on the income tax return because it provides some tax refunds for having children. According to the tax administration, shared/joint custody means that the time children spend in each parent’s home should be “roughly” equal, with no mention of any periodicity, such as every-other-week, for instance.^5^

Furthermore, compared to the usual survey data in which incomes are self-reported and subject to imprecise responses, incomes in tax-income datasets are already filled in by the fiscal administration and are thus definitively more reliable. The complete family composition (number of members and age of children) is also reported.

Since we do not have information on the tax returns about hours worked but indeed do have annual labour market income, we hereafter define the state of “being employed” (versus “not employed”) as receiving annual labour market earnings above at least two months of minimum wage, i.e. 2,100 yearly euros in 2009. We conduct robustness checks on several alternative thresholds and show that our results are not sensitive to the definition used.

## Empirical Strategy

### A Selectivity Issue

Our aim is to assess the effect of shared custody arrangements on the labour market participation of mothers following divorce.

However, the type of post-divorce child arrangement is not random, and couples with shared custody arrangements might be highly selected: they may have different observed and unobserved characteristics They are generally more educated and wealthier (Kitterød & Lyngstad, 2012); they might have less conflictual relationships or more egalitarian values towards sharing parental tasks (Solsona & Ajenjo, 2017); and mothers might be more work-oriented (Walper et al., 2021).^6^ Table 2 shows that mothers who are granted shared custody are more likely to work already before the divorce than mothers who have sole custody. Furthermore, reverse causality might occur if mothers who want to work are granted shared custody arrangements for this reason. Because of this potential selectivity or reverse causality issues, a direct comparison of the two groups of mothers’ outcomes (those with sole custody and those with shared custody) is likely to be biased.

**Table 2:** Mothers’ employment rate before and after divorce, according to child custody arrangements

	Mothers’ employment rate (%)		
	Before divorce	After divorce	
	Mean (se)	Mean (se)	N
Child custody arrangements			
Sole custody	73.5 (44.1)	79.5 (40.4)	51,054
Shared custody	89.5 (30.6)	93.4 (24.8)	9,646
All divorced mothers	75.9 (42.8)	81.5 (38.8)	60,700

French fiscal data, divorcees in 2009. Authors’ calculations

Employment is defined as receiving labour market earnings above two months of minimum wage over a year

### Identification

Finding a way to deal with this selection issue is a challenge. We take advantage of huge territorial discrepancies to correct for the endogeneity of being granted shared custody to estimate a causal effect of child arrangements on women’s labour market participation after divorce.

**Fig. 2:**
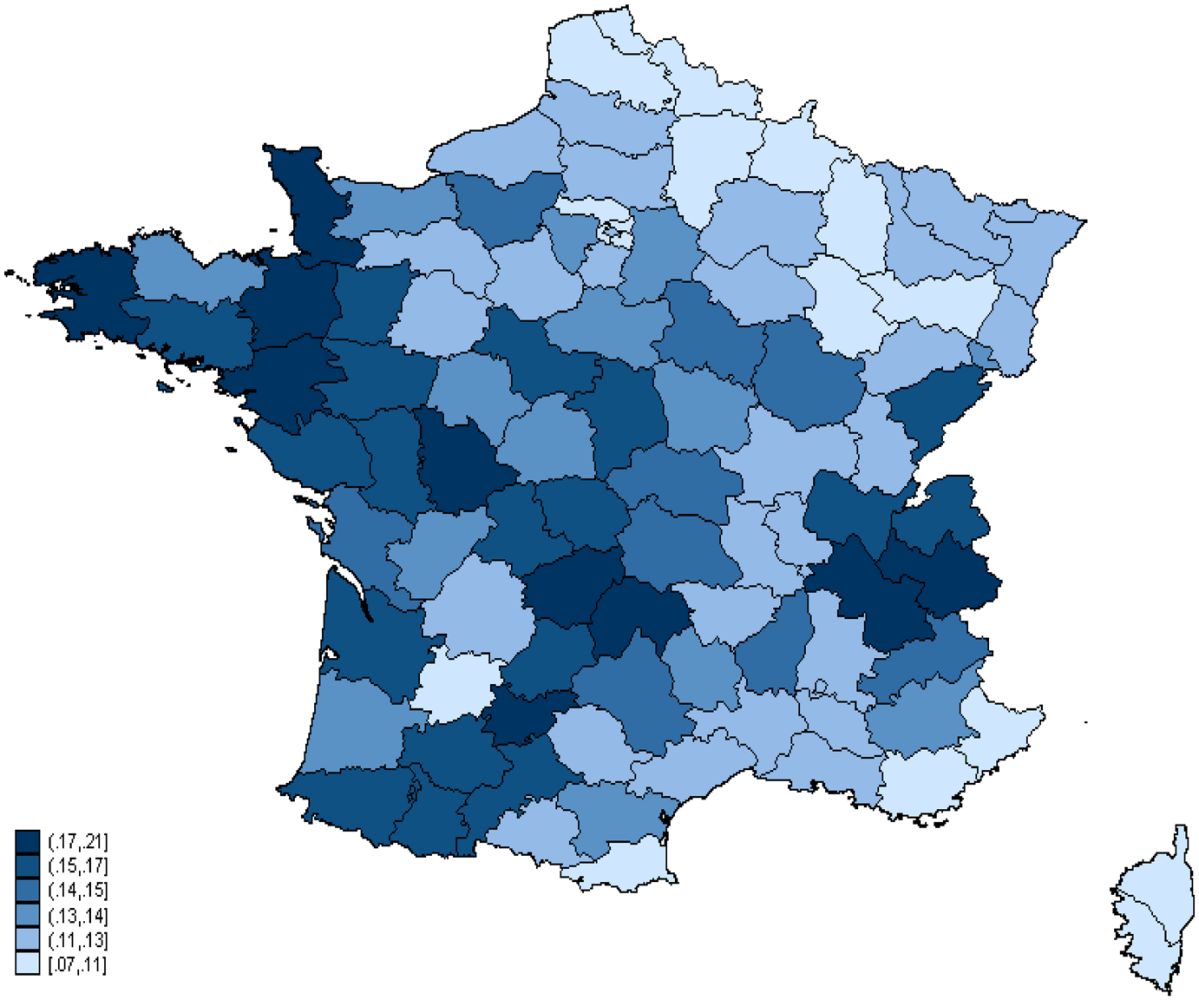
Proportion of shared custody arrangements by French departments (2008**).** Source: Authors’ calculations based on French fiscal data (2008)

Bonnet et al. (2015) and Algava et al. (2019) indeed show that shared custody arrangement decisions in France do not depend only on couples’ characteristics, but also on residential location. The percentage of shared custody arrangement at the county level^7^ in 2008, the year before divorce, ranges from 7 to 21%, depending on the place of residence (Fig. 2).

Custody arrangements are granted as follows. Divorced parents generally follow their lawyer’s advice to decide on child custody arrangements, which they then propose to the family court judge. In the absence of precise guidelines, family judges take their decision by relying on their own individual procedure for considering these different elements while also taking into account the parents’ wishes, as initially advised by their lawyers. The final decision relies on several factors, but mostly on the judge’s opinions regarding the child’s best interests^8^ and on the lawyers’ experience, which altogether may make the final decision exogenous to the initial parents’ choice.

As a proxy for this joint “judge/lawyer effect”, we use the frequency of shared custody arrangements granted in each French county. Since shared custody frequency at the county level may be also influenced by one’s economic situation, religiosity, possibilities for work–family balance, and personal values, we control for many socio-economic and cultural characteristics at the department level and for individual parental characteristics. Once all these dimensions are controlled for, the remaining variability will likely be determined mostly by differences in how diverse judges and lawyers evaluate similar situations. This remaining variability thus constitutes a valid instrument (see also Appendix A2 for additional explanations). Moreover, testing the nullity of the instrument in the first-stage regression results in a high value of the F-statistics, which rules out the risk of a weak instrument issue.^9^

### Models

We first estimate a “naïve” model with a simple probit model on the probability of mothers to be employed after divorce, whether they are granted a shared or sole custody arrangement.

Secondly, as we expect that custody arrangements and labour market participation decisions might be taken simultaneously, we use a simultaneous equation model to take the issue of share custody endogeneity into account. In our context of binary endogenous and dependent variables, we estimate a recursive bivariate probit model to assess the effect of shared custody (SC_*i*_=1 in the case of shared custody) on mothers’ employment (*E*_*i*_ = 1 if the mother is employed).

$$E_{{\text{i}}} = \alpha {\text{SC}}_{{\text{i}}} + \beta X_{{\text{i}}} + C_{{\text{d}}} + u_{{\text{i}}}$$

$${\text{SC}}_{{\text{i}}} = \gamma Z_{{\text{d}}} + \delta X_{{\text{i}}} + C_{{\text{d}}} + \varepsilon_{{\text{i}}}$$

*Z*_d_ is the proportion of shared custody in different departments of France. It is used as an instrumental (exclusion) variable. *X*_i_ includes different pre-divorce covariates at the individual level that could have an influence on having a job: mother’s age (and its square), mother’s pre-divorce activity status, number of children, age of the youngest child, household income quintiles the year before separation, homeownership status, and the size of the town of residence (including a dummy for living in the Parisian region).

*C*_d_ captures potential additional disparities at the department level. First, to take into account the economic situation at the department level, we introduce characteristics of the local labour market, the unemployment rate, and the mother’s employment rate, which also reflects gender norms. As family norms may be important for explaining the level of shared custody, we use information from the Ministry of Labour and Social Affairs Barometer,^10^ a yearly opinion survey, and introduce a regional variable indicating the share of people tending to agree that, ideally, women should stay at home to raise children (compared to people tending to disagree). Second, we control for living conditions with the median fiscal income (in thousands of euros) and poverty rate. As women are more likely to work when the work–family balance is facilitated, we control for the number of places in centre-based childcare per 100 children aged less than 4 years. Finally, we add a dummy for religiosity: the percentage of unaffiliated people in the department (IFOP, 2006). After including a consequent number of potential confounders for the shared custody proportion at the department level, it becomes more plausible to assume that the remaining variability (used as an instrument) is exogenous to parental decisions. We additionally run a regression at the macro-level to see whether the proportion of shared custody at the local level is correlated with these local characteristics (see Appendix A2). This indicates that the proportion of shared custody arrangements at the local level is not related to the factors introduced.^11^

Repartnering may be a potential confounder of post-divorce employment. As Dewilde and Uunk (2008) emphasize, repartnering might be a way to escape poverty and may thus affect labour market behaviours. As repartnering can be potentially endogenous to the choice of post-living arrangements, we deal with it by stratifying the sample and comparing the results of shared custody between two different sub-samples: those who repartner or not during the year following the divorce.

We also provide four additional robustness checks: (1) by estimating a department fixed effect regression, (2) by adding a regional fixed effect (a region is composed of 2 to 8 departments in 2010), (3) by testing the sensitivity of our definition of “being employed”, and (4) by estimating our reduced-form regression on the concerned population and on a placebo population.

## Results

### Baseline Model

The two first columns of Table 3 present the univariate probit model without any correction for the potential endogeneity of shared custody arrangements. The next four columns concern the recursive bivariate probit model for the probabilities of being employed and of shared custody. For each outcome, the coefficients and the marginal effects are displayed.

**Table 3:** Univariate & bivariate probit, whole sample

Variables	Univariate probit employment		Bivariate probit employment			
	Employment		Employment		Shared custody	
	Coef	Marginal effect	Coef	Marginal effect	Coef	Marginal effect
Individual Shared custody	0.300*** (0.028)	0.053*** (0.005)	0.938*** (0.213)	0.144*** (0.028)		
Shared custody per 100 divorces in the department					0.037*** (0.005)	0.008*** (0.001)
Pacs (ref = married)	0.127*** (0.048)	0.024*** (0.009)	0.116** (0.047)	0.023** (0.009)	0.050 (0.033)	0.010 (0.007)
Age	0.098*** (0.011)	0.019*** (0.002)	0.087*** (0.012)	0.017*** (0.002)	0.062*** (0.014)	0.013*** (0.003)
Age^2	− 0.001*** (0.000)	− 0.000*** (0.000)	− 0.001*** (0.000)	− 0.000*** (0.000)	− 0.001*** (0.000)	− 0.000*** (0.000)
2 children (ref = 1 child)	− 0.001 (0.018)	− 0.000 (0.003)	0.001 (0.018)	0.000 (0.004)	− 0.011 (0.018)	− 0.002 (0.004)
3 children	− 0.158*** (0.022)	− 0.030*** (0.004)	− 0.132*** (0.023)	− 0.026*** (0.004)	− 0.159*** (0.022)	− 0.033*** (0.005)
4 and more	− 0.369*** (0.026)	− 0.070*** (0.005)	− 0.320*** (0.033)	− 0.064*** (0.005)	− 0.441*** (0.047)	− 0.093*** (0.010)
Youngest child = 0–3 (ref = 4–12)	0.006 (0.021)	0.001 (0.004)	0.022 (0.020)	0.004 (0.004)	− 0.142*** (0.018)	− 0.030*** (0.004)
Youngest child = 13 +	0.026 (0.022)	0.005 (0.004)	0.060** (0.026)	0.012** (0.005)	− 0.264*** (0.025)	− 0.056*** (0.005)
Household income Q1 (ref = Q3)	− 0.493*** (0.029)	− 0.093*** (0.005)	− 0.440*** (0.042)	− 0.088*** (0.006)	− 0.427*** (0.029)	− 0.090*** (0.006)
Household income Q2	− 0.276***	− 0.052***	− 0.239***	− 0.048***	− 0.208***	− 0.044***
	(0.024)	(0.004)	(0.033)	(0.005)	(0.021)	(0.005)
Household income Q4	0.209***	0.039***	0.165***	0.033***	0.211***	0.044***
	(0.029)	(0.006)	(0.035)	(0.006)	(0.018)	(0.004)
Household income Q5	0.105***	0.020***	0.048	0.010	0.323***	0.068***
	(0.031)	(0.006)	(0.042)	(0.008)	(0.027)	(0.005)
Pre-divorce work	1.199***	0.226***	1.146***	0.229***	0.222***	0.047***
	(0.023)	(0.004)	(0.039)	(0.004)	(0.020)	(0.004)
Owner (ref = renter)	0.149***	0.028***	0.108***	0.022***	0.303***	0.064***
	(0.017)	(0.003)	(0.026)	(0.005)	(0.018)	(0.004)
Rho			− 0.377***(0.144)			
Town size	X		X		X	
Aggregate level controls	X		X		X	
Observations	60,700		60,700			

Clustered (at the department level) standard errors in parentheses

***p < .01, **p < .05, *p < 0.1

Regarding the probability of being granted shared custody arrangement (cols. 5 & 6, Table 3), we observe that our exclusion variable is highly significant. The proportion of shared custody agreements at the local level has a positive and very significant effect on the individual likelihood of being granted a shared custody arrangement. Shared custody is less common for mothers with three or more children than for smaller families, particularly when the youngest child is younger than 4 or older than 12 years old. Older children can decide more freely with whom they wish to live, and shared custody arrangements are less likely for teenagers. We observe an expected positive income gradient in shared custody arrangements. Another indicator of wealth is homeownership status before the divorce, which is positively associated with shared custody arrangements. Shared custody is also less frequent for women who were out of the labour force before the divorce. Consistent with their possibly more traditional values and gender role division, they are more likely to have sole custody after divorce. Lastly, the town size has very little effect.^12^

Regarding women’s employment, being in shared custody arrangements is associated with a 5.3 percentage points higher probability of being employed after divorce (col. 2, Table 3) in the univariate model. After taking into account the potential endogeneity of shared custody (recursive bivariate probit model, col. 4), the effect turns out to be more pronounced. Unobserved selection thus plays a role, as demonstrated by the correlation between the residuals of the two equations, which is negative and significantly different from zero. The probability of being employed turns out to be 14.4 percentage points higher for mothers with shared custody arrangements than for sole custody mothers.

To better understand the direction of the change, as well as the negative correlation between the residuals of the two equations (negative sign of the rho coefficient in the bivariate model), we refer to the usual local average treatment effect (LATE) interpretation. Women who are compliers for our instrument are those who would not have obtained shared custody if they had been in a department that rarely grants it and, instead, they have obtained it because they live in a department where it is more frequently granted. We interpret the negative sign of the rho as follows: women who are compliers (i.e. those who react to the local variation in shared custody) have unobserved characteristics that affect employment negatively. They may be more family-oriented, for instance, than non-compliers.

The other control variables give expected results. The probability of working after divorce increases with the age of the youngest child while it decreases with the number of children. It follows an inverted U-shape curve with a maximum at around age 40. The activity rate is higher for high-income households.

### Heterogeneous Effects

According to their pre-divorce characteristics, being in shared custody arrangements may not have the same labour market consequences for all mothers. To assess heterogeneous effects, we simultaneously interact four main variables (previous occupational status, number of children, age of the youngest child, and household income quintile) with our shared custody arrangement variable (Table 4).

**Table 4:** Shared custody marginal effects on employment (interacted model), whole sample and by repartnering status

	Whole sample	Repartnered	Not repartnered
All mothers	0.218*** (0.017)	0.064 (0.076)	0.237*** (0.011)
By number of children			
1	0.190*** (0.017)	0.042 (0.068)	0.213*** (0.011)
2	0.190*** (0.017)	0.059 (0.066)	0.208*** (0.012)
3	0.275*** (0.018)	0.091 (0.095)	0.293*** (0.011)
4 +	0.411*** (0.017)	0.153 (0.169)	0.424*** (0.012)
By household income			
Q1	0.443*** (0.019)	0.112 (0.160)	0.467*** (0.009)
Q2	0.251*** (0.015)	0.070 (0.098)	0.266*** (0.008)
Q3	0.145*** (0.016)	0.063 (0.044)	0.162*** (0.012)
Q4	0.114*** (0.017)	0.023 (0.036)	0.137*** (0.014)
Q5	0.137*** (0.021)	0.046 (0.042)	0.159*** (0.017)
By pre-divorce work			
working	0.127*** (0.015)	0.045 (0.043)	0.144*** (0.012)
Not working	0.503*** (0.022)	0.123 (0.182)	0.528*** (0.010)
By age of youngest child			
0–3	0.266*** (0.018)	0.081 (0.085)	0.288*** (0.011)
4–12	0.205*** (0.017)	0.068 (0.072)	0.224*** (0.012)
13–17	0.167*** (0.013)	0.024 (0.077)	0.179*** (0.009)
Observations	60,700	13,407	47,293

Clustered (at the department level except for col. 2) standard errors in parentheses. The model includes shared custody variable and all interactions with the listed variables. Only marginal effects are presented

Reading note: If we consider the whole population of divorced mothers, the probability of having a job after divorce in 2010 is 21.8 percentage points higher for mothers with shared custody arrangements compared with those in sole-custody arrangements

***p < .01, **p < .05, *p < .1

Shared custody arrangements play a greater role in women having a job if they were inactive before divorce than if they were already working. The employment rate for mothers who were inactive before divorce and opted for a shared custody arrangement is 50 percentage points higher than for inactive women who had sole custody arrangements. The positive effect of shared custody on female employment is also more pronounced for mothers belonging to the lowest quintile of income before divorce (the probability is 44 percentage points higher for mothers with shared custody than those with sole custody). The benefit of shared custody is less important for mothers who belonged to higher-income households (the probability is 14 percentage points higher for mothers with shared custody than those with sole custody). The positive effect of shared custody arrangements following divorce is also more pronounced for mothers with several children compared to mothers with one or two children. Mothers with infants and in shared custody arrangements are also more likely to work than mothers with infants and their children on almost a full-time basis.

Interestingly, all these results point in the same direction and are fully consistent. Every one of the common penalties encountered by mothers in the labour market remains—having young children, several children, being in a poor household before divorce (possibly associated with a low level of education), career breaks (inactive women) –but are largely reduced in the case of shared custody arrangements after divorce. This means that even though shared custody is more likely to be chosen by wealthier parents and active mothers, and despite having a positive effect on labour force participation for all mothers, we observe more pronounced effects for mothers further away from the labour market. In one sense, this result could be expected, because the women already involved in the labour market have less reason to decrease their labour force participation after divorce, whatever the custody arrangements—especially in a context of decreasing living standards following divorce (Bonnet et al., 2021). However, when looking at women who were further away from the labour market because of their family burdens, marital specialization choices, or human capital, our results show that post-divorce child custody arrangements are crucial for mothers’ employment.

### The Possible Role of Repartnering

We take into account repartnering, which may affect both the labour market and custody arrangement decisions. Forming a new couple may also be endogenous because of selection issues in repartnering as well as potential anticipation effects. Indeed, some studies emphasize that repartnering might be a way to escape poverty (Dewilde & Uunk, 2008). We divide the sample into two subsamples, depending on whether or not divorced women are already in a new relationship within the following year. For women who repartner just after divorce, the type of custody arrangement is no longer significant. Thus, whatever the custody arrangement, the probability of working is the same. However, for women not yet repartnered, the positive effect of shared custody remains and is even more pronounced. Results are very similar to those we previously observed: shared custody has larger positive effects for mothers with several children or with an infant and for those in the lowest income quintiles and are inactive before the divorce.

We interpret the absence of effect of custody arrangement for repartnered women as a way for some women to not only increase their living standards but also diminish the work–family trade-off due to the presence of a stepfather who may take part in childcare.

### Robustness Checks

This section provides several robustness checks. Once we controlled for several variables related to gender norms or local labour market characteristics at the local level (values, religiosity, poverty, employment situation, unemployment, etc.), our identification strategy assumes that the remaining variability in shared custody arrangements is exogenous to these dimensions. However, we cannot be fully sure that we capture all the local characteristics likely to affect both maternal employment and shared custody preference. To go further, we perform two alternative specifications. First, because the observed department variability in shared custody likelihood might be due to unobserved factors other than those already controlled for, we perform an alternative specification by introducing department fixed effects. As such, we control for any heterogeneity at the department level. In this situation, there is no need to use our instrument, since it relies on local variations already captured by our department fixed effects. Second, we introduce in our main specification a local fixed effect at the regional level, which is the geographical level that aggregates several departments. This is another way to control for heterogeneity at the local level, which is less demanding than the first robustness check and allows us to continue using our instrument (and thus assess how its effect changes). Table 5 shows that our previous results are very similar to those obtained with these two new specifications. The magnitude of marginal effects of shared custody on a mother’s employment for all mothers ranges from 22 to 23 percentage points. The magnitudes for different subgroups are very similar whatever the models.

**Table 5:** Shared custody marginal effects on employment (interacted model), Robustness checks

	Main specification	With departement fixed effect	With regional fixed effect
All mothers	0.218*** (0.017)	0.229*** (0.014)	0.219*** (0.017)
By number of children			
1	0.190*** (0.017)	0.200*** (0.014)	0.191*** (0.017)
2	0.190*** (0.017)	0.200*** (0.014)	0.191*** (0.018)
3	0.275*** (0.018)	0.291*** (0.016)	0.277*** (0.019)
4 +	0.411*** (0.017)	0.418*** (0.019)	0.411*** (0.017)
By household income			
Q1	0.443*** (0.019)	0.452*** (0.017)	0.443*** (0.019)
Q2	0.251*** (0.015)	0.273*** (0.014)	0.251*** (0.015)
Q3	0.145*** (0.016)	0.161*** (0.014)	0.146*** (0.016)
Q4	0.114*** (0.017)	0.119*** (0.014)	0.115*** (0.018)
Q5	0.137*** (0.021)	0.139*** (0.016)	0.138*** (0.022)
By pre-divorce work			
Working	0.127*** (0.015)	0.152*** (0.014)	0.141*** (0.016)
Not working	0.503*** (0.022)	0.510*** (0.017)	0.506*** (0.023)
By age of youngest child			
0–3	0.266*** (0.018)	0.279*** (0.016)	0.267*** (0.019)
4–12	0.205*** (0.017)	0.214*** (0.014)	0.207*** (0.018)
13–17	0.167*** (0.013)	0.182*** (0.013)	0.169*** (0.014)
Observations	60,700	60,700	60,700

Clustered (at the department level) standard errors in parentheses. The model includes shared custody variable and all interactions with the listed variables. Only marginal effects are presented

*** p < 0.01, ** p < 0.05, * p < 0.1

Thirdly, we test sensitivity to the definition of labour market participation. In our benchmark estimates, we define employment by considering a threshold corresponding to two monthly minimum wages (2100 euros) earned during the year. To test the robustness of our results, we report in Table 6, the results from using other thresholds corresponding to one (1055 euros: Alternative 1), three (3165 euros: Alternative 2), and four monthly minimum wages (4220 euros: Alternative 3). The results are very robust to these different definitions (Table 6).

**Table 6:** Shared custody marginal effects on employment (interacted model), different definitions of activity

	Def. 1	Main specif	Def. 2	Def. 3
Threshold (in yearly €)	1055	2110	3165	4220
All mothers	0.198*** (0.015)	0.218*** (0.017)	0.229*** (0.018)	0.243*** (0.020)
By number of children				
1	0.176*** (0.015)	0.190*** (0.017)	0.200*** (0.017)	0.2132*** (0.020)
2	0.173*** (0.016)	0.190***(0.017)	0.200*** (0.017)	0.213*** (0.019)
3	0.245*** (0.016)	0.275*** (0.018)	0.289** (0.020)	0.306*** (0.024)
4+	0.370*** (0.012)	0.411*** (0.017)	0.430*** (0.021)	0.453*** (0.025)
By household income				
Q1	0.399*** (0.014)	0.443*** (0.019)	0.469*** (0.025)	0.495*** (0.032)
Q2	0.221*** (0.013)	0.251*** (0.015)	0.272*** (0.017)	0.295*** (0.020)
Q3	0.131*** (0.016)	0.145*** (0.016)	0.151*** (0.015)	0.164*** (0.017)
Q4	0.106*** (0.017)	0.114*** (0.017)	0.116*** (0.015)	0.121*** (0.016)
Q5	0.133*** (0.020)	0.137*** (0.021)	0.136*** (0.019)	0.141*** (0.020)
By pre-divorce work				
Working	0.120*** (0.015)	0.127*** (0.015)	0.125*** (0.014)	0.130*** (0.015)
Not working	0.484*** (0.018)	0.503*** (0.022)	0.517*** (0.028)	0.526*** (0.034)
By age of youngest child				
0–3	0.242*** (0.016)	0.266*** (0.018)	0.279*** (0.019)	0.295*** (0.022)
4–12	0.186*** (0.016)	0.205*** (0.017)	0.216*** (0.018)	0.231*** (0.021)
13–17	0.153*** (0.013)	0.167*** (0.013)	0.175*** (0.014)	0.185*** (0.017)
Observations	60,700	60,700	60,700	60,700

Clustered (at the department level) standard errors in parentheses. The model includes shared custody variable and all interactions with the listed variables. Only marginal effects are presented

***p < 0.01, **p < 0.05, *p < 0.1

Finally, we run the reduced form equation on mothers, for the whole sample of divorced mothers and for the selected sample of divorced mothers who were out of the labour force the year preceding divorce (Table 7). Results show an expected positive sign for the coefficient of the proportion of shared custody at the department level among divorced mothers in both samples. As a placebo test, we repeated this exercise for childless divorced women. This “intention-to-treat” regression enables us to check if the proportion of shared custody at the local level has an effect on childless women who, by definition, are not concerned with shared custody. We did not find any effect of the proportion of shared custody at the local level. This placebo test is an additional confirmation that our instrument is uncorrelated with unobserved characteristics that may play a role in women’s employment after divorce.

**Table 7:** Reduced form and placebo test, probit

	Sample of mothers		Placebo sample: Childless women	
	All	Inactive	All	Inactive
Proportion of shared custody	0.848* (0.450)	1.499* (0.808)	0.231 (0.493)	-0.184 (0.953)
Number of observations	60,700	11,577	18,930	2,802

Controls include women’s age and squared age, PACS, income quintiles, ownership status, unemployment rate, squared unemployment rate, town size, previous activity status (for specifications on the whole sample), number of children, and age of youngest child (for specifications on the mothers’ sample), and aggregate levels variables

*p < 0.1

Were shared custody to have an effect on these women’s employment, it would pose a threat to our identification strategy meaning our instrument may be related to other factors influencing the employment of all women, thus disregarding their custodial arrangements. The placebo test clearly shows that our instrument has no significant effect on childless women: point estimates are between 2 and over 3 times lower than that obtained for mothers, and they are non-significant.

## Discussion and Conclusion

A rapidly increasing trend—both in shared custody practises and in the diversity of parents with shared custody arrangements—is observed in many countries. In the sharp debate about whether or not to promote shared custody arrangements, the main arguments put forth concern either the consequences for children in terms of cognitive and behavioural development, or equality between parents in terms of rights for visitation and equally exercising their parental responsibilities after divorce. The impact of shared custody on labour market outcomes for divorced parents is much less assessed, although it may constitute an important factor in the discussion because it affects the living standards and poverty risk of all family members.

In this article, we analyse women’s labour force participation after divorce, according to child living arrangements. Shared custody significantly increases the probability of lone mothers being employed the year following divorce by 24 percentage points compared to mothers with a sole physical custody arrangement. It may reduce work–family conflicts by reducing childcare expenses and enlarge the possibilities of finding a suitable job due to more relaxed time constraints of lone mothers. Among these large positive effects on mothers’ employment, huge heterogeneous effects are observed for: inactive women; those belonging to the lowest income quintiles before the divorce; those with a young child; and those with three or more children. Shared custody is helpful for women who are far removed from the labour market. Our results are robust to alternative specification of our models and definitions of employment.

The high likelihood of re-entering the labour market after divorce for mothers who were previously out of the labour force is not a new finding (see for instance Finnie, 1993, Thielemans, & Mortelmans, 2019, Bonnet et al., 2021), but the fact that shared custody arrangement enlarge this effect is a new and original contribution.

The causal interpretation of our results is based on the assumption that the remaining local variation in child custody arrangements is exogenous once other possible local specificities (cultural, economic, and social) are taken into account, at least from the family’s point of view. We cannot exclude the possibility that, first, there exist some unobserved factors that go beyond the dimensions we have controlled for and, second, that these may still affect not only the decisions of judges (and lawyers), but also maternal preferences regarding work. Undoubtedly, using instrumental variables is inherently limited and, thus, so are many studies that rely on this strategy. However, several arguments and robustness checks that we provide all point in the same direction. Furthermore, it should be mentioned that our work is the first to attempt using individual information about shared custody arrangements to measure the causal effect of shared custody arrangements on divorced mothers. Vuri’s previous causal study (2018) using child custody law in the US context finds no effect on labour force participation for lone mothers. Since our work finds different results, it warrants replications in other countries, particularly in the current context of increasing shared custody prevalence in many countries. It would be particularly interesting to see whether the causal effects on the behaviours of lone mothers originate from the effective practice of shared custody arrangements or from laws that may possibly be changing parenting norms after divorce.

From a policy perspective, it is interesting to reframe this result in light of the policy against poverty. To fight against poverty, several countries have introduced quite costly welfare programs associated with “welfare to work” and “make work pay” policies, which sometimes specifically target lone parents. The laws favouring joint custody and the increasing trend in this practice are relatively costless from a public policy point of view since parents bear the private costs of maintaining two dwellings with sufficient space for children to live on a regular basis. But the policy may have positive effects on divorced mothers’ labour market outcomes. The extent to which regulations on shared custody might be compared to welfare employment programs constitutes a crucial public policy question and should be seriously considered. Even if child custody arrangements do not fall within the scope of employment policy, our research shows that policies promoting more equal sharing of parental responsibilities—such as those increasing shared custody arrangements—could have strong effects on women’s financial autonomy, at least in the short term, while they could also have potential long-term effects on pension entitlements.

However, our results on the positive effects of shared custody also show that re-entering the labour market after divorce is not universal and is highly sensitive to the financial and time constraints faced by mothers after divorce. In most cases, mothers in France with shared custody arrangements receive either no support payments or a reduced amount from the father, and they benefit from less public support (income tax reductions are more limited than for mothers with sole custody). This is different from the USA, where shared custody arrangements are generally associated with higher support payments. Thus, in France, there might be an incentive for women with shared custody arrangements to work due to the lack of child support payments and less public support in comparison with mothers having sole custody. This probably constitutes higher economic pressure that could affect work behaviour and may explain why our results differ from those of Vuri. Another explanation would be that low-income women have less to lose in shared custody since they are less likely to receive child support payments or smaller amounts even in sole custody. Another highly illustrative example of a post-divorce mother’s constraints is the fact that repartnering may be a way for some women to, first, escape these huge financial constraints (confirming previous studies) and, second, reduce the work–family imbalance. In case of repartnering, the type of child custody arrangement does not play a role anymore.

Although we cannot disentangle time from income mechanisms, we find that mothers of several children and having a young child tend to work more in case of shared custody than in the case of sole custody. This suggests that shared custody may also ease the constraints of child care schedules for divorced mothers.

Our study considers activity status one year after divorce. This is a short period for recovery and constitutes a limitation of our data being available only for this short time frame. Nevertheless, we should expect even stronger effects over time. Finding a job and organizing for child care may take some time, especially for mothers who interrupted their careers before divorce. This work calls for further analysis on both long-term consequences and on fathers’ economic situation since shared custody may have also labour market consequences for them.

Finally, some specifications on the French context can shed light on our results and assess their external validity. France is a family-oriented country, where even mothers with young children work, meaning that this particular country-specific environment provides incentives for mothers to work in the form of quite generous childcare provisions. However, there is an educational gradient in the female employment rate, which is emphasized by recent parental leave policies (Joseph et al., 2013; Lequien, 2012; Piketty, 2005). Mothers belonging to the lowest income quintiles are those more likely to quit the labour market after the birth of a child, and they are more likely to interrupt their careers after the extension of parental leave. Our results for divorced mothers also show that mothers who are furthest away from the labour market are those for whom the type of custody arrangement after divorce is the most important and whose likelihood for employment is more strongly affected. However, instead of a decrease, we observe a higher likelihood of employment. This shows that some specific populations react more than others to either public policies or new family arrangements.

Shared custody in less family-friendly countries is therefore likely to play even more of a role in a mother’s employment, since these mothers receive fewer benefits from public policies designed to balance work and family duties; in which case, our results can be seen as lower bounds on the effect that shared custody has on a mother’s employment. This must be confirmed by replicating our study in other countries, however.

## Data Availability

This paper uses confidential and exhaustive French fiscal data which are only available under some constraints (on a secure server at the National Institute of Statistical studies). We cannot make data publicly available because we are not allowed to share these data.

## Appendixes

### Appendix A1

See Table 8.

**Table 8:** Child support payments received by mother with children in sole custody, by pre-divorce household income quintile

	% receiving child support payments*	Average yearly amount (in euros), whole population	Average yearly amount (in euros), for recipients
Q1	35.8	1023	2901
Q2	50.1	1513	3083
Q3	57.6	1841	3266
Q4	65.8	2704	4198
Q5	71.7	6562	9312

*Child supports payments as declared in tax return by the mother

### Appendix A2: Aggregate Level Regression

As evidence of our instrument’s exogeneity, we present here regressions of the proportion of shared custody at the department level on a set of local controls reflecting the socioeconomic and cultural characteristics of the department. Indeed, the proportion of shared custody may be influenced by the economic situation, religiosity, the possibilities for work–family balance, and personal values.

Only the mothers’ activity rate turns out to have a significant impact on the share of shared custody (Table

**Table 9:** Share of shared custody in the department, aggregate level (OLS regression)

Variables	Coef (se)
Unemployment rate	0.001 (0.015)
Unemployment rate square	− 0.001 (0.001)
Median Income/1000 (in euros)	− 0.003 (0.002)
Poverty rate	− 0.001 (0.001)
Child care places per 100 children under 3	− 0.000 (0.000)
% of the population identifying as atheist (ref = 20–27%)	
Atheist < 20%	− 0.015 (0.010)
Atheist 27–34%	− 0.004 (0.006)
Atheist > 34%	− 0.012 (0.008)
% of individuals with traditional values	0.040 (0.078)
Mothers's activity rate	0.162*** (0.044)
Constant	0.121 (0.108)
Observations	96
Adjusted *R*-squared	0.362

Standard errors in parentheses

****p* < 0.01, ***p* < 0.05, **p* < 0.1

^9^). This is expected, because of the correlation between the individual propensity to work a given year and the global mother’s activity rate during a previous year (although our results remain the same even without this variable). Hence, the local proportion of shared custody is explained only a little by the socio-economic structure or family values. Adjusted R squared is only 36%. There thus remains a lot of unexplained variability that cannot be accounted for by different variables. Of course, we definitely do not claim here that our instrument fully explains the remaining variability, but it clearly rules out the idea that all the local conditions we tested are the main determinants of the local share of shared custody. We think that the local “judge/lawyer effect” is an important part of the story and of this remaining unexplained variability.

## Footnotes

1 In the case of France, from 2002, legal parental responsibilities are shared between parents whether they are married or not. This sharing continues after the couple’s dissolution. Cases where only one parent is granted legal custody (“Autorité parentale”) remain exceptions.

2 Even if in both cases, a part of the expenses for children is incompressible such as housing.

3 See, for instance, articles 373–2-6 and 373–2-9 of the French civil code.

4 The prior-divorce variables used as margins calibration to compute those weights include family structure (age of both partners (or declarant), age and number of children), some housing variables such as localization, the tenure status and the number of rooms, and several income variables: the wage ratio between woman and man, the income tax paid, the taxable income, and the total income reported during all three years preceding the divorce.

5 We cannot completely rule out that – for tax optimization reasons—parents declare child custody arrangements that are different from their actual practices. However, as they are supposed to report the same child custody arrangement on both tax returns, this behaviour is risky and unlikely. As Bonnet et al. (2015) showed, divergences between the two parents are very rare (less than 10%).

6 However, recent works (Meyer et al. (2017) for the US, Kitterød and Wiik (2017) for Norway) emphasize that since shared custody is becoming increasingly common, parents should be thus less and less selected.

7 France is composed of 96 county-like administrative divisions called departments. We do not consider overseas departments.

8 Even when both parents ask for shared custody, judges can refuse it if they consider it can be harmful to the child.

9 To test for a weak instrument issue, we compute the usual first-stage regression, regressing shared custody on our IV and our additional covariates and test for the nullity of the instrument. First, consistent with our nonlinear specification, we use a univariate probit model. The Chi-square statistics obtained for nullity test is 122, which is clearly a high value. Second, to better compare the statistics of the test with the usual thresholds for detecting weak instruments (see Stock et al., 2002), we perform a linear regression. The F-statistic is 76, much higher than the standard thresholds. We are thus confident about the fact that our instrument is not weak.

10 https://drees.solidarites-sante.gouv.fr/sources-outils-et-enquetes/le-barometre-dopinion-de-la-drees

11 With the sole exception of the mother’s female employment rate. However, performing our models with or without mother’s female employment rate does not change our results.

12 Living in very big towns has a small negative effect on having shared custody, which is probably related to the higher price of housing as a constraint to having two dwellings with enough space for children one out of two weeks.
